# Inflammatory Biomarkers in Cardiac Surgery and the Suggestion of an Editors' Heart Team

**DOI:** 10.21470/1678-9741-2018-0607

**Published:** 2018

**Authors:** Paulo Roberto B. Evora, Bruno Pinheiro, Domingo M. Braile

**Affiliations:** 1 Editor-in-Chief Interim - BJCVS Faculdade de Medicina de Ribeirão Preto da Universidade de São Paulo (FMRP-USP), Ribeirão Preto, SP, Brazil; 2 Associate Editor - BJCVS Hospital Santa Genoveva, Goiânia, GO, Brazil; 3 Editor-in-Chief - BJCVS Faculdade de Medicina de São José do Rio Preto (FAMERP), São José do Rio Preto, SP, Brazil and Universidade de Campinas (UNICAMP), Campinas, SP, Brazil

## BJCVS Highlight

Based on the ultrasensitive C-reactive protein (us-CRP) behavior, Abrantes et al.^[1]^ analyzed the inflammation resulting from myocardial revascularization techniques with (on-pump coronary artery bypass grafting - ONCAB) and without (off-pump coronary artery bypass grafting - OPCAB) cardiopulmonary bypass. The authors concluded that there was an increase in us-CRP in the postoperative period compared to the preoperative period. This increase occurred in all moments assessed postoperatively. There was no difference in the us-CRP behavior between the two myocardial revascularization techniques^[1]^.

Despite the unexpected result in differentiating the two coronary artery bypass grafting techniques, the importance of the inflammatory reaction triggered by cardiopulmonar bypass is always a contradictory subject. One has the impression that the issue is overestimated, considering the incidence of systemic inflammatory response syndrome (SIRS) in the milieu of cardiac surgery^[2]^. The search for biomarkers of inflammation is a constant concern, especially considering the cost-benefit ratio. In this regard, cytokine dosage is not justified in clinical and surgical practice.

In the search for markers of the inflammatory reaction, the chemiluminescence dosage of the stable nitric oxide (nitrite and nitrate-NOx) products, although low cost, was not relevant^[3]^. Still taking into account the nitric oxide release pathway, the determination of cyclic guanosine monophosphate (cGMP) could be more sensitive, but the methodology is expensive and difficult to apply in the operating room. Thus, the tendency to search for an inexpensive and easy-to-use biomarker is observed, increasing interest in "old biomarkers" such as C-reactive protein, neutrophil-to-lymphocyte ratio (NLR) and platelet-to-lymphocyte ratio (PLR). This trend may be noted in recent publications in the Brazilian Journal of Cardiovascular Surgery (BJCVS)^[4]^.

NLR is a new addition to the long list of these inflammatory markers. NLR, which is calculated from complete blood count with differential, is an inexpensive, easy to obtain and widely available marker of inflammation, which can aid in the risk stratification of patients with various cardiovascular diseases, in addition to the traditionally used markers. Recently, NLR has been reported as a prognostic marker for the outcome from coronary artery bypass grafting^[5]^.

Despite being a non-specific marker of inflammation, C-reactive protein (CRP) and alkaline phosphatase are routinely measured by hospital laboratories and therefore would be useful in cardiac surgery. Also, neutrophil/lymphocyte (N/L) and platelet/lymphocyte (P/L) ratios have become useful inflammatory biomarkers. Aldemir et al.^[6]^ showed a significant increase in total leukocyte and neutrophil counts and N/L ratio and a decrease in lymphocyte counts were observed at all time points after surgery in both groups. N/L ratio was significantly higher in the with cardiopulmonary bypass (CPB) group compared to the OPCAB group on the first postoperative day, but this difference disappeared on the fifth postoperative day.

Elevated CRP levels have been associated with severe adverse cardiac events, including death. However, the causal association of CRP with atherogenesis is less clear, and there are data suggesting that it is a bystander rather than a true risk factor. It is importante to note that CRP levels decrease in response to anti-inflammatory agents, making it useful for monitoring the efficacy of new anti-inflammatory drugs. The erythrocyte sedimentation rate and CPR analyses are the oldest markers of inflammation and acute myocardial infarction and are still useful in clinical practice^[7]^.

In 1930, Tillet and Francis published the first report on the occasional discovery of CRP. In 1943, the first clues to the possible connection between CRP and atherothrombotic events were described by Lofstrom and later by Kroop^[1]^. Looking at the "eternal pursuit" of credible and economically viable markers, are not we "going back to the past"? The current data is suggestive...

## Heart Team and the Indian Philosophy and the Suggestion of an Editors' Heart Team

The specific term 'Heart Team' is quite recent and was incorporated into European and American College of Cardiology/American Heart Association (ACC/AHA) guidelines subsequent to the pivotal SYNTAX trial. Surely, it gained popularity based on the context of coronary interventions and transcatheter aortic valve replacement (TAVR) and other complex endovascular interventions. However, the 'Heart Team' concept deserves some criticism: 1) The 'Heart Team' can certainly compromise the basis of medical practice based on the doctor-patient relationship; 2) The way we practice the concept of 'Heart Team' today is quite fragmented, inconsistent and uneven with "shades of grey all over"; 3) Unfortunately, there are physicians who can do routine work masquerading as emergency to avoid the 'Heart Team' for doing routine coronary interventions through an emergency route, for better reimbursement and for not putting the patients on the waiting list.

These points are highlighted by Yadava^[8]^: 1) Thinking philosophically, the concept of 'Heart Team' is a reality or is it a 'Platonic Illusion'? 2) The concept of ' Heart Team' in cardiovascular medicine is ahead of its time? 3) The 'Heart Team' may reduce culpability for wrong decisions and subsequent medical-legal litigation; 4) Most of it has been an empty rhetoric, suiting the medical fraternity, but much to the indignation and disadvantage of the hapless patient.

When we chose 'Heart Team' as the opening theme for this edition, in the search for literature data, we find the brilliant text of Yadava^[8]^, which incorporated our ideas one hundred percent. Any attempt to compose the text made clear the plagiarism of ideas.

Dr. Yadava is CEO and Chief Cardiac Surgeon at the National Heart Institute, New Delhi and Editor-in-Chief of Indian Journal of Thoracic and Cardiovascular Surgery. He does research in adult Cardiac Surgery and Cardiology. These personal data led us to the thought, "Why not an Editors Heart Team?" We contacted Dr. Yadava who was ready to write the Editorial published in this issue of the Brazilian Journal of Cardiovascular Surgery (BJCVS). We believe that this provocative text will bring deep reflections.

## Articles in this Issue

This issue of BJCVS presents a blind peer-reviewed selection of 15 papers that were selected by order of acceptance (10 original articles, 2 review articles, and 3 selected case reports). In response to our editorial efforts, five Letters to the Editor have been included in this issue.

**Paulo Roberto B. Evora** ^1^Editor-in-Chief Interim -
BJCVS Faculdade de Medicina de Ribeirão Preto da Universidade de
São Paulo (FMRP-USP), Ribeirão Preto, SP, Brazil**Bruno Botelho Pinheiro** ^2^Associate Editor -
BJCVS Hospital Santa Genoveva, Goiânia, GO, Brazil**Domingo M. Braile** ^3^Editor-in-Chief -
BJCVS Faculdade de Medicina de São José do Rio Preto (FAMERP),
São José do Rio Preto, SP, Brazil and Universidade de Campinas
(UNICAMP), Campinas, SP, Brazil
